# Grey seals use anthropogenic signals from acoustic tags to locate fish: evidence from a simulated foraging task

**DOI:** 10.1098/rspb.2014.1595

**Published:** 2015-01-07

**Authors:** Amanda L. Stansbury, Thomas Götz, Volker B. Deecke, Vincent M. Janik

**Affiliations:** 1 Sea Mammal Research Unit, School of Biology, University of St Andrews, Fife KY16 8LB, UK; 2 Centre for Wildlife Conservation, University of Cumbria, Nook Lane, Ambleside, Cumbria LA22 9BB, UK

**Keywords:** anthropogenic noise, acoustic fish tags, dinner bell effect, chemosensory cues, pinnipeds

## Abstract

Anthropogenic noise can have negative effects on animal behaviour and physiology. However, noise is often introduced systematically and potentially provides information for navigation or prey detection. Here, we show that grey seals (*Halichoerus grypus*) learn to use sounds from acoustic fish tags as an indicator of food location. In 20 randomized trials each, 10 grey seals individually explored 20 foraging boxes, with one box containing a tagged fish, one containing an untagged fish and all other boxes being empty. The tagged box was found after significantly fewer non-tag box visits across trials, and seals revisited boxes containing the tag more often than any other box. The time and number of boxes needed to find both fish decreased significantly throughout consecutive trials. Two additional controls were conducted to investigate the role of the acoustic signal: (i) tags were placed in one box, with no fish present in any boxes and (ii) additional pieces of fish, inaccessible to the seal, were placed in the previously empty 18 boxes, making possible alternative chemosensory cues less reliable. During these controls, the acoustically tagged box was generally found significantly faster than the control box. Our results show that animals learn to use information provided by anthropogenic signals to enhance foraging success.

## 1. Introduction

Most studies on the effects of anthropogenic noise primarily consider negative impacts on animals [1–4]. These effects can be pronounced, such as lethal beaked whale strandings coinciding with exposure to military sonar [5]. More commonly, increased noise levels can impact local abundance and distribution, damage auditory organs, increase stress, change vocalization behaviour and lead to hypertension, decrease reproductive success and mask other biologically relevant sound sources [1–4].

While the detrimental effects of noise have been relatively well investigated, comparatively little research has considered how increased noise can be beneficial to animals. There are several ways animals can exploit increased noise levels; masking by anthropogenic noise can protect prey from acoustic detection by predators [1] or conversely increase foraging success of predators by preventing acoustic detection by prey [6]. Western scrub jays (*Aphelocoma californica*) prey upon eggs of nesting species, but avoid areas with increased noise [7]. Thus, noise pollution can decrease nest predation and therefore increases reproductive success of prey species [7]. Such benefits of noise may explain the increased success of some birds in habitats with intensive human activity [8,9].

The use of sound from a localized acoustic source can also facilitate learning by indicating a location of interest. Acoustic deterrent devices (ADDs) aim to elicit avoidance responses in aquatic predators, such as seals, and are currently being used to reduce depredation in fisheries. However, seals that have previously found fish at a location close to an ADD quickly habituate to these sounds [10,11]. Observational evidence suggests that ADDs may also attract predators [12] and in such cases may even cause higher incidences of predation [13] due to contextually learned associations between sound and prey, also known as the ‘dinner bell’ effect. If sounds introduced by humans regularly serve as such a signal, it may influence animal behaviour and ecology much more widely than previously assumed.

We tested this experimentally by studying the behaviour of seals exposed to acoustic fish tags. Such coded acoustic tags are used widely to monitor marine fish and invertebrates [14]. The tags produce ultrasonic frequencies which are assumed to be imperceptible to the marked animal. However, while the target species may not be sensitive to the tag signal, the signal was predicted to be audible to some predators including seals [15]. In an experimental study, sensitivity thresholds for a Vemco 69 kHz fish tag signal were measured and used to estimate detection distances for a harbour seal (*Phoca vitulina*) and California sea lion (*Zalophus californianus*). Both species were found to be capable of detecting the signal (source level of 165 dB re 1 µPa) at simulated distances of greater than 200 m [16].

While this past work has shown that seals are capable of perceiving fish tag signals, it is unknown whether they use this information for prey detection. Here, we examine whether naive grey seals (*Halichoerus grypus*) learn to use sounds from fish tags as an ‘acoustic beacon’, potentially making tagged fish more vulnerable to predation.

## 2. Material and methods

### (a) Subjects

Ten juvenile grey seals (three females, seven males), born on the Isle of May (Firth of Forth, Scotland), were the subjects of this study. Four of the seals were born in November 2011 and six in November 2012. The seals had been followed from birth, had never been in the sea, and had no previous experience associating sound with food. After weaning (at approximately three weeks of age) pups were transferred to the licenced captive facility at the Sea Mammal Research Unit (St Andrews, Scotland). All seals were of very similar age when taken into our facility. At the start of testing, they were approximately three months old and were tested over a period of six months. The seals were fed a varied diet of several fish species, however during testing only whole adult herring (*Clupea harengus*, approx. 100 g in size) was used. Seals were released back into the wild within 1 year of initial capture.

### (b) Testing enclosure

**Figure 1.**
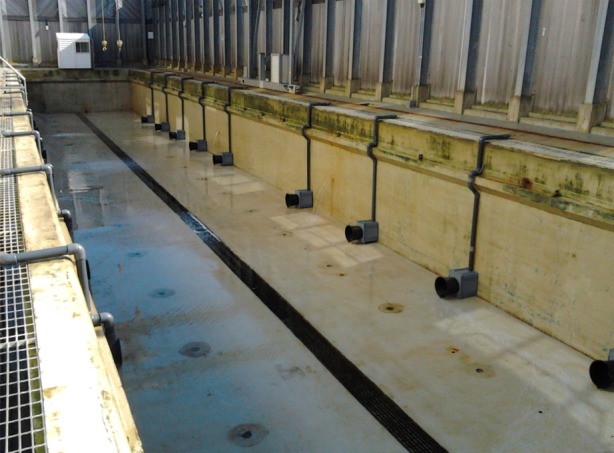
Photograph of the testing enclosure, drained of water. Twenty foraging locations were distributed around the pool. At each location, the seal could place its head through a bucket to access fish hidden within the box. (Online version in colour.)

The seals were tested in a 37.5 × 6 × 2.25 m concrete pool. Twenty foraging locations were equally distributed around the bottom of the pool (figure 1). Each foraging spot consisted of a PVC pole suspended from the surface with a chamber at the base. The chamber consisted of a 35 × 30 × 40 cm box, a 25 × 34 cm bucket and a 14 × 16 cm door flap (electronic supplementary material, figure S1). The fish were hidden inside the box; to retrieve a fish, the seals could put their heads into the bucket and through the door flap. The bucket allowed the seal's head to enter the box, but restricted how far into the box the seal could reach. From within the box, the fish could then be taken from a plate where it was secured with an elastic band. The fish were placed either on the plate, where the seal could reach them, or below the plate, where the seal could not access the fish. Magnetic reed switches on the door flap and the plate holding the fish interfaced with a customized Matlab program which logged door-opening and fish removal events. The program recorded the location and time to the nearest millisecond.

### (c) Desensitization, training and testing procedure

Typically an acoustic bridge (a sound signal paired with primary reinforcement such as food) is used for animal training. To ensure our seals were not biased towards the fish tag due to a learned association between sound and food, the seals where not exposed to an acoustic bridge, nor was any other sound associated with food while in our care outside of the experiments reported here.

The seals were initially reluctant to approach the test boxes. Consequently, each seal was given a desensitization period where they were free to access and take fish from a single box. This desensitization occurred in a separate, adjacent pool to where test trials occurred. Each seal retrieved 10 fish from this single box before the experiment began.

**Figure 2.**
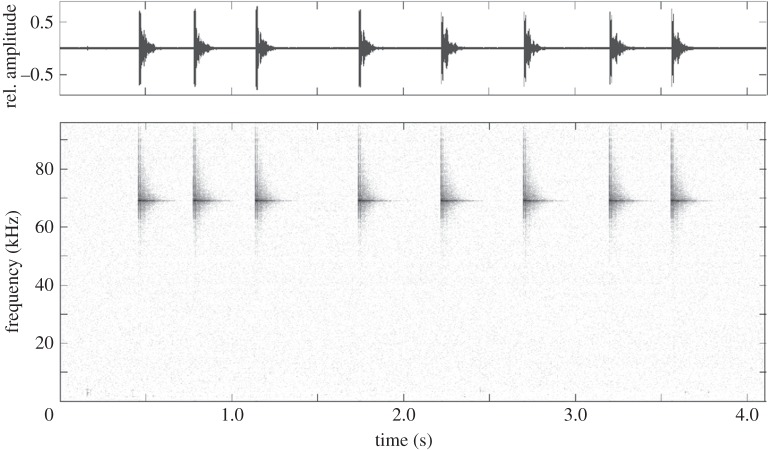
Waveform and spectrogram display of a Vemco V9 coded fish tags, emitting an intermittent 8-pulse, 69 kHz signal (source level 151 dB SPL re 1 µPa).

In the learning experiment, each seal was released into the 20 box array where two pseudo-randomly chosen boxes contained a fish. During each trial, the tagged and untagged fish were placed into two separate boxes, balanced such that throughout the course of the 20 trials every location was baited once with a tagged and once with an untagged fish. One of these, the ‘tagged fish’ treatment, also contained two Vemco V9–2H coded fish tags which emitted an intermittent 69 kHz signal (source level 151 dB SPL re 1 µPa, figure 2). Each signal consisted of an 8-pulse emission unique to each tag (interval between pulses ranged from 0.25 to 0.6 s), which on average resulted in a tag signal in the pool every 13 s (s.d.: 8 s, measured over a 1 h period). To monitor the tag signal, all sessions were audio recorded using a Lumbertek TS150 hydrophone and Edirol R44 recorder (sampling rate 192 kHz, 24 bit). The other box only contained a fish without tags and did not emit any sound. The seal was free to visit and revisit the boxes in any order. When the seal retrieved the tagged fish, the tags stayed in the box and continued to emit signals until the trial ended except in 18% of all trials in which the reed switch was set to turn off one or both of the tags after the fish was retrieved. Turning off tags only occurred at the start of our experiments when we did not know how seals would react to continuing tag sounds. Once we saw no reactions to the continuing tag signals, we left tags active after fish retrieval. The trial was ended by removing the seal from the test pool, either 5 min after the seal had found both fish or after 1 h if both fish were not found. Nine seals took part in 20 of these learning trials, while one animal had only 19 trials. Each seal took part in up to eight trials per day, with successive trials being a minimum of 15 min and maximum of 48 h apart.

Initial results from the four animals tested in 2011 showed that the seals found both the tagged and untagged fish faster and with fewer box visits across the learning period. This suggested that the seals were at least partly using alternative cues, such as chemoreception, to locate fish during the learning experiment. Hence, two additional control experiments were conducted. The first ‘tag only’ experiment consisted of two trials where acoustic tags were placed in one of the 20 boxes, but no fish was placed in any box. As no fish were in the pool, this eliminated any possible chemosensory cue. All 10 seals took part in these ‘tag only’ trials. The second ‘all fish’ experiment was carried out with the six seals studied in 2012 and consisted of two trials where additional fish pieces (inaccessible to the seal) were placed in the previously empty 18 boxes, so that each box contained either a whole accessible fish or a piece of fish that was inaccessible at the start of the tests. Similarly to the learning experiment, only two fish were accessible to the seal (one tagged fish and one untagged fish), the position of which were randomized for each trial. For inaccessible fish, the seals could still reach into the boxes with their heads, but could not reach the fish piece. Between trials the tagged and untagged fish were replaced, while the inaccessible fish pieces were reused. During the second trial, the inaccessible fish pieces were relocated from the new accessible fish boxes to the previously used accessible fish boxes. Thus in trial 1 all fish (both accessible and inaccessible) were new, while in trial 2 the accessible fish were new while the inaccessible fish were reused. Both control experiments occurred with a maximum delay of 2 days after each individual completed the learning experiment. Trials in all control experiments had a maximum inter-trial interval of 20 min. In 2012, when seals went through both kinds of controls (the ‘tag only’ and ‘all fish’ trials), the two controls were conducted a minimum of 15 min and a maximum of 48 h apart.

### (d) Analysis

If the acoustic signal emitted by the fish tag was used as a signal for prey detection, the seals should have found the tagged fish in less time and with fewer box visits than the untagged fish. During the learning period in which the association between the tag and fish was made, the time and number of box visits to finding the tagged fish should have decreased across trials. As the tags emitted sound intermittently and at random intervals, the inconsistent signal may have made the box difficult to localize. Hence, the number of repeat box visits for any of the three box types (box with untagged fish, box with tagged fish and all other boxes containing neither fish nor tag) per trial was used as an additional response variable.

Data were analysed using generalized linear mixed effects models (GLMMs) [17,18]. GLMMs are recommended as an analysis tool where data are non-normal and variance is caused by random effects [17]. In our dataset, the distributions of all response variables were non-normal but could be well modelled with a Poisson (repeat visits, number of boxes visited) or gamma distribution (time models). The generalized form of the mixed model allowed us to predict coefficients on the scale of the response variable without the need for transforming raw data [18]. Random effects manifested themselves in variation in a subject's response to the treatment across trials [17]. The use of a mixed model meant that we could account for this variation without having to model a large number of fixed effects (covariates) or having to ignore this variance entirely as would be the case with simple hypothesis testing. The inclusion of a crossed random effect between individual and trial number in the candidate models also allowed us to account for the repeated measures design of the study (subjects were tested repeatedly in consecutive trials). Models were fitted using the glmer function in the lme4 package (1.0–5) for R 3.0.1 [19]. The models all included at least box type as a fixed effects factor and subject as a random effects factor. Additionally, trial number and box distance from the position where the seals entered the pool as well as the interaction term of box type and trial number were considered as covariates.

Models were fitted to the data of each of the three different experiments. For the learning experiment (20 trials), we created a model to predict time taken to find the fish (with a gamma error distribution and logarithmic link function), a model to predict the number of boxes visited before retrieving the fish (Poisson error distribution and logarithmic link), and a model to predict the number of repeat visits by box type throughout the 20 learning trials (also with a Poisson error distribution and log link function). The log link was chosen for the model with a gamma distribution (time to fish model) because candidate models using the canonical (inverse) link did often not converge during the iteration process. The gamma models that did converge with the canonical link provided similar results to the ones with the log link. Offset terms were considered for the model on the number of repeat visits to the boxes. Trial length was not included as an offset term in the repeat visit model as it did not show a correlation with the number of box visits. However, an offset term consisting of the number of boxes of a specific type present in the experiment was included (i.e. one box each with a tagged and untagged fish, 18 empty boxes). For the control experiments, we created two models to predict time to fish retrieval, one for each of the two control conditions (‘tag only’ or ‘all fish’ trials) each fitted using a gamma error distribution and logarithmic link function.

In cases where a seal failed to find either the tagged or untagged fish and therefore no time to fish retrieval or number of box visits before fish retrieval could be measured, all observations within the trial were excluded from analysis in the corresponding model. This happened in up to 9% of trials for each seal. Additionally, for some trials a door switch malfunctioned so that no count for the number of box visits could be obtained. If a fish box door switch failed (19 trials in a total of 199 trials), the data for that box were excluded from the analysis.

A stepwise model selection procedure was carried out using a second-order Akaike information criterion (AICc) [18]. Firstly, the ‘beyond optimal model’ with the interaction term (and additional covariate) was specified and different random effects combinations were tested. The tested combinations (here shown in R notation) were a random intercept term for subject (1 | subject) and random slope terms for trial number and box type within subject (trial number | subject) and/or (box type | subject). The selected random effects in the final models were (trial number | subject) for the ‘time to fish’ models and (trial number | subject) + (box type | subject) for the ‘number of box visits' model and the ‘number of boxes visited before retrieving the fish’ model. Secondly, the optimal combination of fixed effects was determined. Tested fixed effects included trial number and its interaction with box type and distance from the point where the seal entered the pool to the fish boxes as a potential additional covariate (for the ‘time to fish’ models only). The interaction term of box type (tag presence) and trial number would indicate a learning effect, i.e. the seal finding the tagged fish faster towards the end of the 20 learning trials. In one case, a candidate model for a control experiment did not converge and had to be excluded from the selection process. In the box visit model, contrasts between the three levels of the factor box type were tested using the lsmeans function from the lsmeans package in R [20]. The fixed effects combinations retained in the final selected models are shown in tables 1–3.

**Table 1.** Generalized linear mixed effects models for the time and number of boxes visited before finding the tagged and silent fish during the 20 learning trials (gamma distribution and log link). Model coefficients (effect size) for fixed effects are presented on the scale of the response variable. Significant (*p* < 0.05) variables are highlighted in italics.

	model	coefficient e^β^	CI		*p*
			2.5%	97.5%	
(intercept)	time	480.72	235.33	981.98	<*0.0001*
	box visits	20.671	16.354	26.128	<*0.0001*
box type	time	1.245	0.738	2.101	0.412
	box visits	0.971	0.789	1.195	0.783
trial number	time	0.915	0.882	0.949	<*0.0001*
	box visits	0.957	0.940	0.975	<*0.0001*
distance	time	1.014	1.001	1.026	*0.0382*
	box visits	1.020	1.017	1.023	<*0.0001*
box type × trial number	time	0.963	0.923	1.004	0.0792
	box visits	0.982	0.973	0.991	*0.0001*

**Table 2.** Generalized linear mixed effects model for the number of repeat box visits during the 20 learning trials (Poisson error distribution and log link). Model coefficients for fixed effects are presented as incident ratios on the scale on the response variable. Significant (*p* < 0.05) variables are highlighted in italics.

	coefficient e^β^	CI		*p*
		2.5%	97.5%	
(intercept)	2.749	2.469	3.060	**<***0.0001*
acoustic tag with fish	2.390	2.256	2.530	**<***0.0001*
fish only	1.430	1.331	1.535	**<***0.0001*

**Table 3.** Generalized linear mixed effects models for the time to finding the tagged versus untagged fish box during the two control conditions (gamma distribution and log link). Model coefficients for fixed effects are presented on the scale of the response variable. Significant (*p* < 0.05) variables are highlighted in italics. ‘nr’ indicates the variable was not retained in the model selection process.

	model	coefficient e^β^	CI		*p*
			2.5%	97.5%	
(intercept)	all fish	249.6	56.678	1099.232	<*0.0001*
	tag only	66.511	38.644	114.474	<*0.0001*
box type	all fish	0.011	0.003	0.044	<*0.0001*
	tag only	0.464	0.341	0.632	<*0.0001*
trial number	all fish	0.221	0.086	0.568	*0.0017*
	tag only	2.090	1.413	3.089	*0.0002*
distance	all fish	1.085	1.053	1.119	<*0.0001*
	tag only	nr	nr	nr	nr
box type × trial number	all fish	8.637	3.688	20.226	<*0.0001*
	tag only	nr	nr	nr	nr

The final model assumptions were checked using diagnostic plots of model residuals. This procedure revealed one residual which was an extreme outlier that could disproportionately influence the overall outcome of the ‘time to fish’ model for the learning experiment. To test the effect of this residual, the model was refitted without the outlier and these results are presented in the electronic supplementary material. Confidence intervals (CIs) were calculated using Wald statistics and both model parameter coefficients and CIs are shown on the scale of the response variable.

## 3. Results

**Figure 3.**
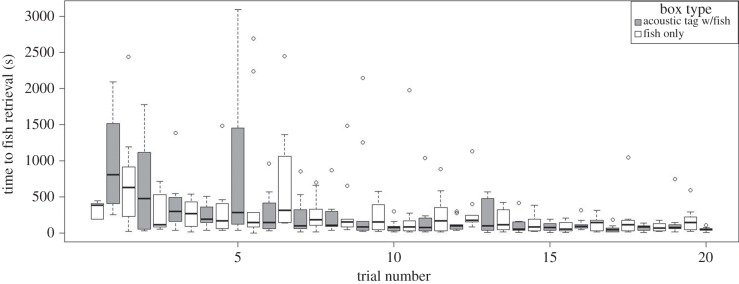
Tukey's boxplots for the time to finding fish, either with or without the fish tag, by trial throughout the learning period (*n* = 10 seals for trial 1–19, *n* = 9 seals for trial 20).

**Figure 4.**
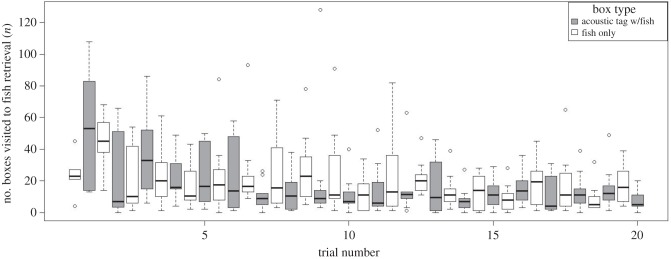
Tukey's boxplots for the number of boxes visited before finding fish, either with or without the fish tag, by trial throughout the learning period (*n* = 10 seals for trial 1–19, *n* = 9 seals for trial 20).

**Figure 5.**
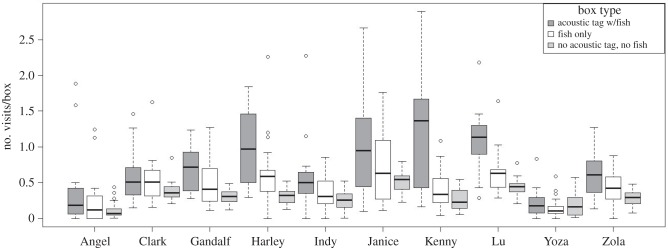
Tukey's boxplots for the number of repeat box visits by individual seals by box type, either fish with or without the fish tag or empty box, in the learning experiment. Pairwise difference obtained from model contrasts are shown above the graph. ***p < 0.0001. (*n* = 20 trials for each seal except for Yoza, who only had 19 trials).

In the 20 trial learning experiment, time and number of boxes visited to finding fish decreased across training trials, showing a learning curve (figures 3 and 4). The mixed model showed a highly significant effect of trial number with a reduction in time and number of boxes visited before retrieving the fish over consecutive trials (GLMM, table 1). The GLMM also showed that seals needed less time and fewer box visits to find fish in boxes that were closer to the pool entrance. The interaction term of tag presence and trial number was highly significant for number of boxes visited before fish retrieval. This indicates that seals visited fewer boxes before finding the acoustically tagged fish compared with the untagged fish in later trials. However, there was no consistent effect of box type (‘tagged’ or ‘untagged’) on time needed to finding the fish (large CI, table 1). This suggests that seals either used alternative sensory cues (most likely through chemoreception) to locate fish from untagged boxes and perhaps even from tagged boxes during the learning experiment or that the animals increased their swimming speed when looking for food from trial to trial. The interaction term of box type (presence of the tag) and trial number approached significance in the standard model and became highly significant in the model when a single extreme outlier was removed (electronic supplementary material). This significant interaction indicates that seals needed approximately 5% less time to find the box that contained the tag with each consecutive trial. Additionally, the seals revisited the box with the tagged fish most frequently (figure 5). The mixed model (GLMM) for repeated box visits (table 2) showed that seals visited boxes that initially held the untagged fish 1.4 times more often than empty boxes. However, the acoustic tag caused a 2.4-fold increase in the number of repeat visits compared with empty boxes. Seals revisited boxes with the acoustic tag significantly more often than boxes that initially contained untagged fish as revealed by highly significant contrasts between the three levels of the factor box type (*p* < 0.0001).

**Figure 6.**
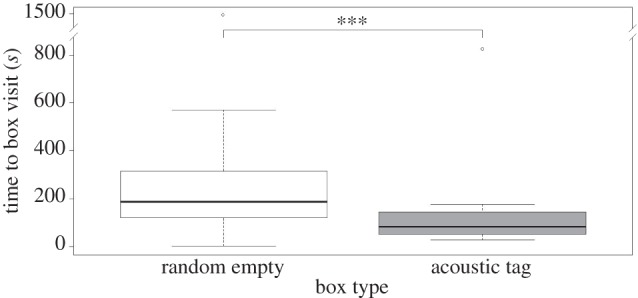
Tukey's boxplots for the time to finding the ‘tagged’ box compared to a randomly selected empty box for the two ‘tag only’ control trials in which no fish was placed in any box while acoustic tags were placed in one box (*n* = 10 seals, two trials each).

In the first control experiment (the ‘tag only’ trials), no fish was placed in any box while one box contained acoustic tags. Time to finding the acoustic tag box was compared to that of a randomly selected box. The results differed markedly from the learning experiment as tag presence caused a significant reduction (approx. 54%) in time needed to visit the box (GLMM, table 3 and figure 6), confirming the seals learned to use acoustic cues in the 20 initial trials. While tag presence reduced the time needed to find the acoustic tag box across both trials, the time needed to visit a box was twice as high in the second trial compared with the first (GLMM, table 3).

**Figure 7.**
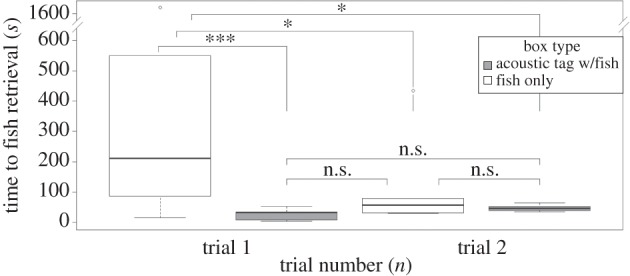
Tukey's boxplots for the time to finding the fish, either with or without the fish tag, for the two ‘all fish’ control trials in which fish were placed in all 20 boxes, but were only accessible from two of the boxes. Model contrasts are shown above the graph with ***p < 0.0001, *p < 0.01 and n.s., not significant (*n* = 6 seals for each trial).

These results were supported in the second control experiment (the ‘all fish’ trials), in which chemosensory cues were made unreliable by placing fish in all boxes, with only two of these fish being accessible to the seals. The model showed that tag presence caused a significant reduction in time needed to retrieve the fish (GLMM, table 3 and figure 7). The interaction between trial number and tag presence was also significant, showing the effect differed between the first and second trial of this experiment. The model contrasts that show the significant differences between the pairings are presented in figure 7. Seals needed less time to find the tagged fish in trials 1 and 2 when compared with finding the untagged fish in trial 1. It also becomes obvious that seals needed less time to find the silent fish in the second trial than to finding it in the first trial of the all-fish controls which may indicate the ability to differentiate chemosensory cues from fresh versus reused fish. Seals also found the fish near the pool entrance faster than those further away (table 3).

## 4. Discussion

Our study documented the use of novel environmental cues by grey seals during a foraging task. Seals dramatically reduced the time and number of boxes visited to find fish by adapting their foraging behaviour to use environmental information within a relatively short-time period (within 20 trials). The significant interaction between trial number and box type shows that the seals found the tagged fish in fewer box visits than the untagged fish in later trials, demonstrating the learned use of the acoustic tag to locate food. While seals did not find the tagged fish much faster than the untagged fish during this learning experiment, there is evidence for a weak interaction effect between trial number and presence of the acoustic tag. These results indicate that animals may have used chemosensory cues as a primary source of information to locate fish during the learning experiment, but gained additional information from the tag in later trials, or that they increased their overall searching speed.

The use of the acoustic tag is additionally supported by the increased number of visits to tagged boxes, demonstrating that seals learnt the relevance of the acoustic cues and adjusted their foraging strategy to revisit profitable foraging spots. While the seals may have initially revisited tagged boxes as an exploration of a novel stimulus (the tag), this would have been expected to decrease with experience. However, there was no difference in repeated box visits across trials (not retained in model selection). Additionally, this increase of visits to the tagged box location was stronger than to the untagged fish location, showing that animals did not just return to a previously successful foraging site but that they were influenced by the continuing acoustic signal. These results are particularly relevant when considering the potential impact of long-term sound sources, such as net pingers or ADDs. The seals were 2.4 times more likely to re-visit a tagged location than any other empty box, despite the tagged box remaining depleted (table 2). This effect was significant despite a number of initial trials in which the tag did not emit signals after fish retrieval (see Material and methods).

The effect of the tag became particularly apparent in the control experiments where chemosensory cues became unreliable due to either no fish being placed in the boxes (‘tag only’ trials) or the presence of fish in all boxes (‘all fish’ trials). In these experiments, the presence of the acoustic tag caused significant reductions in the time needed to find a fish. In the ‘tag only’ trials, the tagged box was found faster in the first trial, and it took twice as long to find in the second trial. This may be due to an extinction effect, where exposure to the tag without a fish reduced the animal's response in the second trial. Interestingly, in the ‘all fish’ trials, the seals also managed to reduce the time needed to find the untagged fish in the second trial, suggesting either a chemosensory ability to distinguish older from more recent baits since inaccessible fish were not changed between trials or a motivational or practice effect that led to an added focus on the other boxes in the second trial.

Our findings present a novel way of looking at anthropogenic noise that illustrates how animals exploit cues when they become available. It is difficult to assess the extent to which seals could rely on such acoustic and chemosensory cues present in this experiment when foraging in the wild. Live, mobile fish are likely to provide less chemosensory information than the dead fish used in our captive experiment, which could make acoustic fish tags in the wild a more dominant and reliable cue. However, the movement of live fish together with the low duty cycle of acoustic tags may make acoustic signals less efficient. The acoustic signal from a tag may be most beneficial to a predator when emitted from sedentary and inconspicuous prey where hydrodynamic swim trails that can be used for prey detection may be less obvious [21–23]. Detection range may impact which cues are most salient; acoustic tags may increase prey detection from a distance by attracting experienced seals to locations with mobile tagged fish, where they then use other sensory inputs for prey capture. Our results therefore illustrate the importance of considering the auditory sensitivities of all animals in the environment when designing an acoustic tagging study for a selected species. The learned association between a signal and food leading to a ‘dinner bell’ effect has been demonstrated in several species. Other marine animals are similarly capable of using noise information and associative learning. This effect may be most pronounced in marine mammals with low auditory thresholds in high-frequency bands. Detection ranges for 69 kHz tag signals in odontocetes, for example, have been predicted to exceed 1 km [15].

Acoustic fish tags are being used extensively in mark–recapture studies to assess fish survival [24–27]. Research agencies worldwide invest significant resources in acoustic tagging studies to assess fish stocks and determine survival rates. As acoustic tags could make a fish more vulnerable to predation, tagging can lead to erroneous conclusions in such studies. This concern is supported by observations of decreased survivorship rates for acoustically tagged juvenile salmon compared with those with similar tags that produce no sound signal [26–29]. Similarly, tagged predator species may experience a decrease in foraging success. Acoustic tags are becoming more widely used on sharks [30–32] and could make them more detectable by prey species such as seals [16]. Even recently published reports of acoustically tagged seals meeting at sea [33] could be caused by a tag attraction effect, as the tags used produced sounds similar to the fish tags used in the area. In the case of the seals, possible solutions to reduce detectability of tags include an increase in the frequency of the tags as well as increasing the onset time in case detectability is primarily due to the onset click. Such tags are currently commercially available. However, care should be taken as other predators with higher frequency sensitivity, such as cetaceans, could still detect such tags.

All tagging studies rely on the basic assumption that tags have no significant impact on marked individuals. However, our results suggest that acoustic tags could have profound effects on the fitness of the studied individuals in situations where they are audible to conspecifics, predators or prey. Similar tag effects have been widely investigated in the use of rings to mark birds; ring colour and symmetry alters mate selection, reproductive success [34–38] and dominance interactions [39]. Marking also increases detectability by predators; tadpoles marked with a skin staining dye are more susceptible to predation than unmarked tadpoles [40]. While most research has examined the effects of visual marking, here we showed that acoustic tags comparably aid prey detection, potentially increasing predation of tagged animals. Acknowledging such impacts of marking, both for visual and acoustic tags, is critical to research generalizing the behaviour and mortality of marked animals to natural populations.

Past research has focused on detrimental physiological effects of noise on animal fitness, and taken little consideration for how anthropogenic noise may be used by some organisms to increase foraging success. Artificial noise sources are widely deployed in various anthropogenic activities or in an attempt to study or manipulate animal behaviour. Examples include the fish tags tested here, but other acoustic devices such as net pingers, echosounders, boat engines, turbines, sonar and ADDs could be similarly exploited for beneficial information. This may explain the direct approach route that some seals take when approaching foraging spots around wind farms [41]. Thus, when introducing artificial sound sources into an environment, it is important to take into consideration all potential effects on local species, both detrimental and beneficial.

We demonstrated that anthropogenic signals can be used to an animal's benefit as a signal to detect prey. Similar results could be expected for many animal species that can perceive noise signals. A shift in foraging behaviour as demonstrated here can have profound effects on an ecosystem. Future studies need to focus on the relevance of such modifications in foraging interactions to assess their wider implications.

## Supplementary Material

Stansbury_et_al_ESM

## Data accessibility

Data from this study are available at the British Oceanographic Data Centre (http://www.bodc.ac.uk/).
