# Supplementary material for: Grey seals use anthropogenic signals from acoustic tags to locate fish: evidence from a simulated foraging task
Source: Proc Biol Sci. 2015 Jan 7;282(1798):20141595. doi: 10.1098/rspb.2014.1595 (PMC4262164; doi:10.1098/rspb.2014.1595)
Supplement: Stansbury_et_al_ESM [file rspb20141595supp1.pdf]

Supplemental Material

Figure S1

Photograph of the foraging boxes showing the view from the A) front of the box, B) side of the box and C) inside of the box. To access food, seals placed their head into the bucket, and through a door flap to take fish secured on a plate with elastic band. Time when the door flap opened or closed and when the fish was taken was recorded by automatic switches.

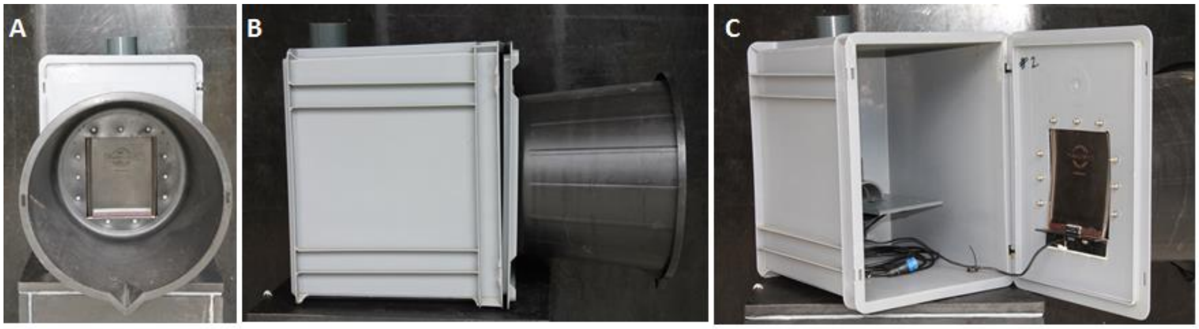

Table S1

Results from the generalized linear mixed effects models (GLMM) for the time before finding the tagged and untagged fish during the 20 learning trials with the outlier removed (gamma distribution & log link). Significant ($p<.05$) variables are highlighted in bold. Model coefficients for fixed effects are presented on the scale of the response variable.

	coefficient	confidence interval		p
	e^{β}	2.5%	97.5%	
(intercept)	445.8	231.42	858.87	**<0.0001**
acoustic tag (box type)	1.363	0.860	2.160	0.188
trial #	0.914	0.882	0.950	**<0.0001**
distance	1.018	1.007	1.030	**0.001**
acoustic tag * trial #	0.949	0.914	0.985	**0.006**
